# The Provision of Texture-Modified Foods in Long-term Care Facilities by Health Professionals: Protocol for a Scoping Review

**DOI:** 10.2196/44201

**Published:** 2023-03-17

**Authors:** Dianis Wulan Sari, Gading Ekapuja Aurizki, Retno Indarwati, Farapti Farapti, Etty Rekawati, Manami Takaoka

**Affiliations:** 1 Department of Advanced Nursing Faculty of Nursing Universitas Airlangga Surabaya Indonesia; 2 Research Group for Community Health, Family, and Geriatric Nursing Universitas Airlangga Surabaya Indonesia; 3 Division of Nursing, Midwifery and Social Work School of Health Sciences The University of Manchester Manchester United Kingdom; 4 Department of Nutrition, Faculty of Public Health Universitas Airlangga Surabaya Indonesia; 5 Department of Community and Geriatric Nursing Faculty of Nursing Universitas Indonesia Depok Indonesia; 6 Department of Gerontological Home Care and Long-Term Care Nursing The University of Tokyo Tokyo Japan

**Keywords:** aged, diet, dysphagia, health professional, texture-modified foods, residential care

## Abstract

**Background:**

Malnutrition among older adults with dysphagia is common. Texture-modified foods (TMFs) are an essential part of dysphagia management. In long-term care (LTC) facilities, health professionals have implemented TMFs, but their application has not been fully elucidated, making them heterogeneous.

**Objective:**

We aim to explore the implementation of TMFs in LTC facilities, particularly focusing on the role of health professionals in nutritional care involving TMFs (eg, deciding the type of food, preparing and giving the food, and evaluating the outcomes).

**Methods:**

A scoping review using the PRISMA (Preferred Reporting Items for Systematic Reviews and Meta-Analyses) methodological approach will be performed. A comprehensive search for published literature will be systematically performed in PubMed, CINAHL, MEDLINE, ProQuest, PsycINFO, and Science Citation Index (Web of Science). Data screening and extraction will be performed by 2 reviewers independently. The studies included will be synthesized, summarized, and reported, following the preferred reporting items of the Mixed Methods Appraisal Tool. Our review will consider the following study designs: mixed methods, quantitative, and qualitative. Studies with patients who are not older adults will be excluded.

**Results:**

Data extraction will be completed by February 2023. Data presentation and analyses will be completed by April 2023, and the final outcomes will be completed by June 2023. The study findings will be published in a peer-reviewed journal.

**Conclusions:**

Our scoping review will consider studies related to TMF interventions for older adults in LTC residential facilities, with no exclusion restrictions based on country, gender, or comorbidities. Studies on interventions that address TMF-related issues, such as deciding the type of food, preparing and giving the food, and evaluating the outcomes, are qualified for inclusion.

**Trial Registration:**

OSF Registries 79AFZ; https://osf.io/79afz

**International Registered Report Identifier (IRRID):**

PRR1-10.2196/44201

## Introduction

### Background

Malnutrition is prevalent among the older adult population. The prevalence of malnutrition among older adults has been reported to be between 1.3% to 47.8% in the community [1], but in long-term care (LTC) facilities, the prevalence of malnutrition is much higher, ranging from 30% to 60%, and malnutrition has a significant negative impact on the health of older adults [2-4]. Malnutrition in older adults is often associated with dysphagia [5]. The prevalence of dysphagia among older adults ranges from 12% to 60% [6-8]. Texture-modified foods (TMFs) are commonly prescribed for older adults to address the problem of dysphagia [9,10].

Although standardized terminology and international terminology for TMF have been established [11-13], the implementation of TMF is still reported as a public health concern and has resulted in adverse events [14-16]. The implementation of TMF in LTC facilities is related to many factors, including health professionals' knowledge, health facility financing, infrequent dysphagia screening, and the misunderstanding of TMF calories. To understand the gap in TMF implementation, the exploration of TMF implementation by health professionals needs to be reviewed to understand future directions.

Previous studies have reported on the implementation of TMF in LTC facilities [17-19]. However, relatively little research that explores complete information on TMF implementation by health professionals in LTC facilities has been conducted [20]. TMF implementation, the frequency of giving TMFs, target patients, the types of TMFs used, and the implementers of TMF in LTC facilities need to be explored to improve TMF implementation and identify research gaps in TMF implementation within LTC facilities.

An international framework for the implementation of TMF has been developed [13,21]. However, there is no existing literature review on TMF implementation in LTC facilities [22]. There are no clear rules regarding the implementation of TMF in LTC facilities, how to implement TMF, and who should implement TMF. We conducted a preliminary search of PubMed, and no current or underway scoping reviews and systematic reviews on the topic were identified.

The objective of our scoping review is to explore the implementation of TMF in LTC facilities, particularly focusing on the role of health professionals in nutritional care involving TMF.

### Review Questions

Our scoping review will address the following research question, mainly focusing on the role of health professionals in nutritional care involving TMF (eg, deciding the type of food, preparing and giving the food, and evaluating the outcomes): how is the implementation of TMF in LTC facilities?

### Participants

Our scoping review will consider studies describing the implementation of TMF in LTC residential facilities. Older adults will be defined in our study as those aged ≥60 years [23]. No exclusions will be made based on country, gender, or a specific medical condition.

### Intervention

Health professionals have implemented TMF in LTC facilities. Health professionals undertake remunerated work for which formal education is required (eg, nurses, dietitians, physicians, speech therapists, and paramedical workers). They are classified based on the International Standard Classification of Occupation [24,25]. Studies will be excluded if the roles of health professionals in the provision of TMF are not clearly defined. We aim to explore the implementation of TMF in LTC facilities, mainly focusing on the role of health professionals in nutritional care involving TMF (eg, deciding the type of food, preparing and giving the food, and evaluating the outcomes).

### Concept

The concept of interest is any implementation of TMF. TMFs and thickened drinks are commonly used to reduce choking risks and aspiration [26]. Studies on the implementation of TMF will be considered eligible if they explain who implemented TMFs in LTC facilities; how to implement TMF; and at least one of the following: how to decide the type of TMF, how to prepare and give TMFs, how to improve nutritional support in geriatrics through TMFs, how to evaluate the implementation of TMFs, or how to address any other TMF-related issues. Our study will include older adult–reported outcomes and health professional–reported outcomes described by the authors. Studies will be eligible if they include at least one TMF-related implementation.

### Context

We will select studies set in LTC facilities. LTC facilities are designed institutions that provide formal (paid) accommodation and include health or social LTC facilities [27]. Studies will be included if their contexts suit this LTC facility description. Studies will be included regardless of the registration, orientation, type, or scope of the LTC facilities or the qualifications of health care professionals. Studies will be excluded if patients were cared for without health care professionals working in LTC facilities. Studies will also be excluded if institutionalized care was conducted as home care. Studies conducted in diverse facilities will be included if findings from LTC facilities can be identified [28].

### Types of Sources

Mixed methods, quantitative, and qualitative study designs are eligible for inclusion in our review. We will include studies published from database inception to the date of the search. Documents will be considered eligible only if the full text is available. Only articles available in English, Japanese, and Bahasa Indonesia will be considered for inclusion due to limited resources that do not allow for the screening and translation of studies in other languages. The search process will include a filter for participants aged ≥60 years.

## Methods

### Overview

The final scoping review will be reported based on the PRISMA-ScR (Preferred Reporting Items for Systematic Reviews and Meta-Analyses Extension for Scoping Reviews) [29]. A preliminary search was carried out on Google Scholar on September 3, 2022, using keywords (*texture modified food*, *health professional*, and *long term care*). There is no existing review that addresses a similar aim as that of our review. Our scoping review is registered in Open Science Framework (registration number: 79AFZ).

### Search Strategy

The comprehensive search of bibliographic databases will use PubMed, CINAHL, MEDLINE, ProQuest, PsycINFO, and Science Citation Index (Web of Science). Our search strategies will use the following process [30,31]. First, the scoping review protocol constructs will be identified with an initial logic grid that aligns with the participants, intervention, concept, and context of interest. Second, a pilot search of 2 databases—PubMed and Web of Science—will be conducted. An author will analyze the titles and abstracts of retrieved papers and then confirm the index terms used to describe the articles. Third, the initial logic grid will be reorganized, using all identified keywords and index terms for all included databases (Textbox 1). Fourth, the databases will be screened in the last search for potentially relevant records that were missed in the previous search. The full texts of retrieved review articles will also be reviewed to identify additional potentially relevant documents. An example of the search strategy is presented in Table 1. The pilot search was completed between August and September 2022.

**List of search terms.**

**Search terms related to participants**

- *Older adults, Elderly, Aged, and Senior*

**Search terms related to intervention**

- *Health professionals; Personnel, Health; Health Care Providers; Health Care Provider; Provider, Health Care; Healthcare Providers; Healthcare Provider; Provider, Healthcare; Healthcare Workers; Healthcare Worker; Health Care Professionals; Health Care Professional; and Professional, Health Care*

**Search terms related the concept**

- *Texture modified foods, Texture modified, Texture food, and Texture modified diet*

**Search terms related to the context**

- *Long-term care, Care residential facilities, Residential care, Residential facilities, Residential care institutions, Nursing homes, Elderly homes, Care homes, Assisted living facilities, and Skilled nursing facilities*

**Table 1:** Example of the search strategy for PubMed (date of search: September 2022).

Search query number	Query	Records retrieved, n
1	*((((older adults)) OR (elderly)) OR (Aged)) OR (Senior)*	5,963,158
2	(((((((((Long$term care) OR (Care residential facilit*)) OR (Residential care)) OR (Residential facilit*)) OR (Residential care institution*)) OR (Nursing home*)) OR (Elderly home*)) OR (Care home*)) OR (Assisted living facilit*)) OR (Skilled nursing facilit*)	464,315
3	(((Texture$food*) OR (Texture$diet)) OR (Texture$modified)) OR (Texture modified food*)	12,282
4	Query 1 *OR* query 2	6,178,105
5	Query 4 *AND* query 3	1145
6	(((((“Health professional*”) OR (“Health personnel”)) OR (“Health$Care Provider*”)) OR (“Health Care Professional*”)) OR (“Healthcare Worker*”)) OR (“Health Care Professional”)	315,421
7	Query 5 with the following filters: English, Indonesian, and Japanese	1064
8	((((((older adults)) OR (elderly)) OR (Aged)) OR (Senior)) OR ((((((((((Long$term care) OR (Care residential facilit*)) OR (Residential care)) OR (Residential facilit*)) OR (Residential care institution*)) OR (Nursing home*)) OR (Elderly home*)) OR (Care home*)) OR (Assisted living facilit*)) OR (Skilled nursing facilit*))) AND ((((Texture$food*) OR (Texture$diet)) OR (Texture$modified)) OR (Texture modified food*)) (filters: English; participants aged ≥65 years, ≥80 years, and >80 years; and middle-aged participants [ie, 45-64 years])	783
9	((((((older adults)) OR (elderly)) OR (Aged)) OR (Senior)) OR ((((((((((Long$term care) OR (Care residential facilit*)) OR (Residential care)) OR (Residential facilit*)) OR (Residential care institution*)) OR (Nursing home*)) OR (Elderly home*)) OR (Care home*)) OR (Assisted living facilit*)) OR (Skilled nursing facilit*))) AND ((((Texture$food*) OR (Texture$diet)) OR (Texture$modified)) OR (Texture modified food*))	1173

### Study Selection

Reference management software (Mendeley [Elsevier]) will be used to manage the retrieved sources and identify duplicate references. The full-text version of an article will be reviewed if the article’s eligibility is unclear based on the abstract. Two independent reviewers will screen the full texts of selected studies based on the inclusion criteria. The exclusion of references will be recorded and reported in the scoping review. Any disagreements between reviewers will be resolved through discussion or with the help of a third reviewer.

### Data Extraction

The extraction of qualitative data will be carried out in 2 stages. First, the reviewers will read the studies and identify different opinions in the paper, such as original statements from the study participants (ie, first-order constructs) and their interpretations by the researchers or authors (ie, second-order constructs) [32]. Second, the reviewers will reread the studies to extract all raw data. The Excel (Microsoft Corporation) database program will extract the papers included in the review process (Multimedia Appendix 1). The Excel data will include information about the characteristics of the publications, residential facilities, older adults, and health professionals; the implementation of TMF; and the assessment of TMF implementation. The data extraction form will be modified if needed and detailed in the full scoping review. Further, 2 researchers will independently extract the data, 2 researchers will confirm the consistency of the extraction, and 1 reviewer and 1 verifier will review all papers. Any disagreements among reviewers will be resolved through discussion. If a paper has missing data or needs additional data, we will contact the authors of the paper. Such papers will be omitted or described as papers that are missing data if the papers’ authors do not respond in 14 days.

### Data Presentation

A thematic analysis approach will be used to synthesize the data. The data synthesis will consist of the following three stages: (1) synthesizing, (2) summarizing, and (3) reporting the papers reviewed. The stages will follow the preferred reporting items of the Mixed Methods Appraisal Tool [33,34]. A synthesis of analytical themes will be performed. Appropriate information will be reported for our conclusions, including study types, aims, characteristics, types of providers, types of recipients, and information on the implementation of TMF. The study will provide descriptive data (eg, number of studies included, types of study designs, and characteristics of study populations). The implications of the study for future research priorities will be defined.

## Results

Data extraction will be completed by February 2023. Data presentation and analyses will be completed by April 2023, and the outcomes will be completed by June 2023. The main results of our investigation will be presented in a narrative form, focusing on research results to date regarding TMF implementation by health professionals. Additional data on publication years, countries, study designs, populations, and settings will be presented in diagrams or in tabular format.

## Discussion

### Anticipated Findings

The results from our scoping review will explore the implementation of TMF in LTC facilities, particularly focusing on the role of health professionals in nutritional care involving TMF (eg, deciding the type of food, preparing and giving the food, and evaluating the outcomes). Health professionals play a vital role in TMF-related intervention and decision-making within LTC facilities. To our best knowledge, our study will be the first review of the implementation of TMF in LTC facilities, and it will mainly focus on the role of health professionals in nutritional care involving TMF.

We propose that the results of our study will support the development of existing feeding policies in LTC facilities, especially by identifying current knowledge on the role of health professionals in TMF implementation. Several literature reviews have been performed regarding TMF [22,35,36], including a recent review that focused specifically on the unique challenges in LTC.

The implementation of TMF is tightly related to the quality of care in LTC facilities. Older adults' safety while eating is an essential part of standard safety care in LTC facilities [37]. However, choking incidents in LTC facilities still have low levels of attention, although they may be recognized and understood by health professionals [38]. TMF is provided clinically to reduce choking risk among older adults [13,22,39]. This knowledge may help to improve the design and implementation of future TMFs in LTC facilities, so that their benefits become clear and valuable to older adults.

A limitation of our study might be the concepts of LTC facilities among countries and papers. The country-specific terminology about LTC facilities that has been used relates to goals, providers, activities, and target groups [27,40,41]. Comparing types of LTC facilities is beyond the aim of our study. Therefore, our scoping review will define *LTC facilities* as designed institutions that provide formal accommodation and health or social LTC services for older people.

### Conclusion

To our knowledge, our review will be the first systematic scoping review to provide knowledge about the implementation of TMF in LTC facilities, particularly focusing on the role of health professionals in nutritional care involving TMF. Our study will address the evidence-practice gap regarding nutritional sustenance in LTC facilities. The policy makers, decision makers, and health professionals in LTC facilities have insight on TMF implementation. This knowledge may help to improve the design and implementation of future TMFs in LTC facilities. The review will also seek to identify further research gaps and the possible need for further study on TMF.

## Appendix

Multimedia Appendix 1 The proposed data extraction form.

Multimedia Appendix 2 Peer-review reports in Indonesian and English from the Kementerian Pendidikan, Kebudayaan, Riset, Dan Teknologi - Universitas Airlangga - Lembaga Penelitian Dan Pengabdian Masyarakat / Institute for Research and Community Service (Surabaya, Indonesia).

## Abbreviations

LTC: long-term care
PRISMA: Preferred Reporting Items for Systematic Reviews and Meta-Analyses
PRISMA-ScR: Preferred Reporting Items for Systematic Reviews and Meta-Analyses Extension for Scoping Reviews
TMF: texture-modified food

## Data Availability

All data that have been generated or analyzed during the study are included in this published research protocol (and its supplementary information files). The main outcome analyses are being conducted at the time of publication of this protocol, but specific data sets might be available from the corresponding author upon reasonable request.
