# Supplementary material for: The Provision of Texture-Modified Foods in Long-term Care Facilities by Health Professionals: Protocol for a Scoping Review
Source: JMIR Res Protoc. 2023 Mar 17;12:e44201. doi: 10.2196/44201 (PMC10131749; doi:10.2196/44201)
Supplement: Multimedia Appendix 1 [file resprot_v12i1e44201_app1.docx]

| **Multimedia Appendix 1: The proposed data extraction form** |  |
| --- | --- |
| **Characteristics of the publication:** |  |
| Title: |  |
| Author(s): |  |
| Year: |  |
| Type of paper: |  |
| Research method: |  |
| **Characteristics of the residential setting:** |  |
| Country (ies): |  |
| Type of residential facility (e.g., LTC, nursing home): |  |
| Other characteristics: |  |
| **Older adults and Health professional characteristics:** | |
| Older adults’ characteristics (e.g., age, health condition): |  |
| Health professional characteristics (e.g., age, type of health professional, role in the implementation): |  |
| **Implementation of texture modified food:** |  |
| Purpose of implementation: |  |
| Type and description of implementation: |  |
| Provider(s) of implementation: |  |
| Use guideline for implementation: |  |
| Specific role of health professionals (deciding type of food, preparing, giving, and evaluating the outcomes): |  |
| **Assessment of the implementation:** | |
| Sample size and characteristics: |  |
| Implementation outcome(s): |  |
| Assessment of the outcome(s): |  |
| Main findings: |  |
| **Comments or other relevant data:** |  |
