# Supplementary material for: The Provision of Texture-Modified Foods in Long-term Care Facilities by Health Professionals: Protocol for a Scoping Review
Source: JMIR Res Protoc. 2023 Mar 17;12:e44201. doi: 10.2196/44201 (PMC10131749; doi:10.2196/44201)
Supplement: Multimedia Appendix 2 [file resprot_v12i1e44201_app2.pdf]

Hasil Komen Reviewer dan Penilaian Hibah Riset Internal

No	Pengusul	NIP	Judul proposal	Kelompok Riset	Skema Hibah	Reviewer 1	Score	Reviewer 2	Score	Total Score	Dana Yang disetujui
11	**(Dianis Wulan Sari)**<br>Retno Indarwati<br>Farapti	198805062017123201<br>197803162008122002<br>198104142008122001	The provision of texture modified foods in long-term care facilities by health professionals: A scoping review	Keperawatan Gerontik<br>Nutrisi	Riset Mandat: Artikel Review	• Isu yang akan dikaji bersifat essensial dan sesuai dengan bidang keilmuan tim peneliti.<br>• *Roadmap* penelitian rinci dan berkualitas.<br>• Relevansi penelitian dengan kata kunci SDGs belum ditulis.<br>• Tahapan protokol review telah dijelaskan secara detail dan baik.<br>• Ketua pengusul hibah penelitian memiliki rekam jejak yang baik dalam publikasi internasional terindeks kuantil 1 di Scopus (Q1).<br>• Tim peneliti berasal dari lintas fakultas sesuai dengan keahliannya di bidang gizi.	65	• Tema proposal penelitian ulasan ini sangat membantu untuk penelitian selanjutnya.<br>• Peneliti menjelaskan dengan baik bagaimana pertanyaan penelitian akan dijawab dengan metode yang direncanakan, misalnya: menjelaskan jenis artikel yang akan dimasukkan dan dikecualikan, penilaian bias dan kata kunci yang diuji.<br>• Metode dan alat analisis telah dijelaskan dan dilampirkan dengan baik.<br>• Jika studi dilanjutkan pada penelitian asli di masa depan, maka berpotensi menghasilkan produk yang dapat dikembangkan di masa depan.	50	67	Rp. 49,060,000,-

KEMENTERIAN PENDIDIKAN, KEBUDAYAAN, RISET, DAN TEKNOLOGI
UNIVERSITAS AIRLANGGA
LEMBAGA PENELITIAN DAN PENGABDIAN MASYARAKAT
Kampus C Mulyorejo Surabaya 60115 - Telp. (031) 5995247 Fax. (0315923584)
laman : http://lppm.unair.ac.id; e-mail : penelitian@lppm.unair.ac.id, pengmas@lppm.unair.ac.id

No : 2327 /UN3.15/PT/2022 November 21, 2022
Subject: Summary of peer-reviewed

To whom it may concern

Dear Sir/Madam,

On behalf of the Institute of Research and Community Service, Universitas Airlangga, Indonesia. As a grant agency for the Internal Research Grant of Universitas Airlangga, we confirmed that a grantee research proposal was peer-reviewed by two independent reviewers. The selected research proposal has a higher score.

Title of Project	:	The provision of texture modified foods in long-term care facilities by health professionals: A scoping review
Research team	:	Dianis Wulan Sari, Retno Indarwati, Farapti, Gading Ekapuja Aurizki
Scheme of Grant	:	Article Review Research Grant, Internal Research Grant of Universitas Airlangga
Research Group	:	Geriatric Nursing and Nutrition
Faculty	:	Faculty of Nursing and Faculty of Public Health
Total Budget	:	IDR 49,060,000,- (approximately USD 2,750)

KEMENTERIAN PENDIDIKAN, KEBUDAYAAN, RISET, DAN TEKNOLOGI
UNIVERSITAS AIRLANGGA
LEMBAGA PENELITIAN DAN PENGABDIAN MASYARAKAT
Kampus C Mulyorejo Surabaya 60115 - Telp. (031) 5995247 Fax. (0315923584)
laman : http://lppm.unair.ac.id; e-mail : penelitian@lppm.unair.ac.id, pengmas@lppm.unair.ac.id

The summary of peer-reviewed protocols and grant proposals in detail as follows:

Reviewer 1	Score	Reviewer 2	Score	Total score
• The issue that will be reviewed is essential and by the scientific field of the research team.<br>• The research roadmap is detailed and qualified.<br>• The relevance of the research to the SDGs keyword has not yet been written.<br>• The stages of the review protocol have been explained in detail and well.<br>• The head of the research grant proposer has a good track record in international publications indexed quantile 1 in Scopus (Q1).<br>• The research team came from across faculties according to their expertise in nutrition.	65	• The theme of this review research proposal is helpful for the following study.<br>• The researcher explains well how the research question will be answered with the planned method, for example: explaining the types of articles that will be included and excluded, assessment of bias and the keywords that have been tested.<br>• The analytical methods and tools have been well explained and attached.<br>• If the study is continued on original research in the future, it has the potential to produce products that can be developed in the future.	50	67

Best Regards,
Head of Institute Research and Community Service,
Universitas Airlangga

Dr. Gadis Meinar Sari, dr., M.Kes.
NIP 196605041996032001
